# Lithemic Naso-Pharyngitis Due to Systematic Disturbance

**Published:** 1905-02

**Authors:** 


					﻿Lithemic Naso-Pharynoitis Due to Systematic
Disturbance.*
This paper was read by the author at the last annual meeting of
the Association and elicited considerable favorable discussion.
The author treats of the class of cases who are, as he says, “a
miserable, snorting, snuffling, handkerchief-using, irritable and
uncomfortable set who blame all their ills on ‘catarrh? ”
Very commonly no fault can be found locally to explain the ill
nature of the nasal passages. These cases are closely related to
rheumatism and the theory is advanced that excess of indican often
found in the urine is the most patent excitant of the nasopharyn-
geal irritation.
Incorrect habits of diet lie at the base of the difficulty. More
or less constipation exists, little water is drunk, meat and sweets
are freely used as well as salads, coffee, acids, and tea. The skin
is florid and muddy.
Stopping of the autointoxication from imperfect intestinal ac-
tion will therefore cure these cases.
The treatment consists of high colon flushing with soap suds,
followed by the injection of eight ounces of warm olive oil a few
hours later. The patient is ordered to rest in bed until the oil has
acted, which usually takes ten hours.
That putrefaction of products in the intestine occurs and can
cause a poisoning of the system, the author feels is certain, and
scybalous masses not uncommonly pass from such a patient for
three weeks after starting the olive oil treatment.
The diet is of course regulated—acid fruits are forbidden—ex-
ercise in the open air and free use of drinking water between meals
is encouraged.
♦By Dr. J. H. Stucky, (Journal American Medical Association, October 15, 1004).
				

